# Solitary fibrous tumor of the orbit presenting in pregnancy

**DOI:** 10.4103/0301-4738.49405

**Published:** 2009

**Authors:** Jayanta K Das, Angshuman Sen Sharma, Akshay Ch Deka, Dipankar Das

**Affiliations:** Sri Sankaradeva Nethralaya, Beltola, Guwahati, Assam - 781 028, India

**Keywords:** Computer tomography, immunohistochemistry, orbitotomy, pregnancy

## Abstract

A 32-year-old woman, three months pregnant, reported with the complaint of protrusion of the right eye for six months. She gave history of rapid protrusion of eyeball for the last two months along with the history of double vision for the last one month. Computer tomography (CT) scan revealed a well-defined mass lesion in the intraconal space of the right orbit which was excised through a lateral orbitotomy approach. Histological examination and immunohistochemistry revealed a solitary fibrous tumor, which showed a rapid progression in pregnancy.

Solitary fibrous tumor (SFT) is a rare spindle-cell neoplasm usually found in the pleura but has been recently described in extra-pleural sites including the orbit. We report an orbital SFT presenting in a 32-year-old lady with rapid progression during pregnancy.

## Case Report

A 32-year-old woman, three months pregnant, reported with the complaint of protrusion of the right eye for six months, which had progressed rapidly for the last two months. She also gave history of double vision for the last one month. Extraocular movements were grossly restricted in the right eye. Anterior segment examination was unremarkable. The intraocular pressure measured with applanation tonometer was 26 mm Hg in the right eye and 14 mm Hg in the left eye. Visual acuity (Snellen chart) in the right eye was 20/120, N12 and 20/20, N6 in the left eye. There was an axial proptosis of 14 mm in the left eye. Posterior segment examination revealed a few choroidal folds in the posterior pole of the right fundus.

**Figure 1:**
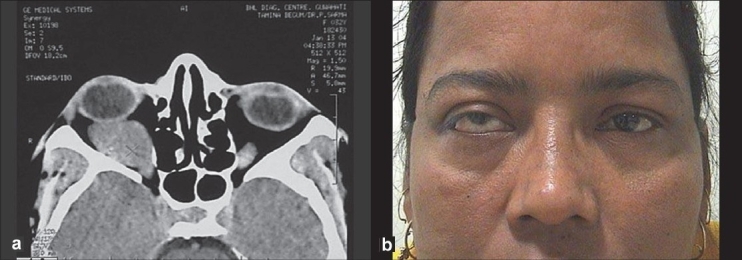
(a) CT scan of the right orbit (axial view) showed an intraconal mass lesion of the right orbit (b) clinical view of the patient (preoperative)

Computer tomography (CT) scan revealed a well-defined mass lesion in the intraconal space of the right orbit, which measured 37 mm × 25 mm × 22 mm, with globe wall flattening in the posterolateral aspect [Fig. 1a, 1b]. The optic nerve was displaced superomedially. CT scan impression was suggestive of cavernous hemangioma (right orbit). Considering her hyperglycemic status (fasting blood sugar 148 mg/dl, post prandial blood sugar 270 mg/dl) and amenorrhea, opinion from obstetrician and endocrinologist was sought.

**Figure 2:**
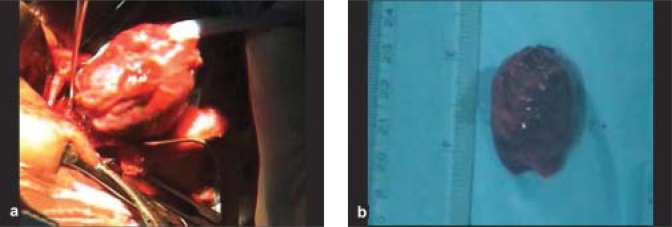
(a) Intra-operative view of the mass during lateral orbitotomy (b) gross appearance of the excised mass

As per the advice of her obstetrician, she underwent medical termination of pregnancy (MTP), due to her high-risk obstetric history, (twice post caesarean section, poorly controlled hyperglycemic state) and because the pregnancy was the result of a failed contraception. Following MTP and normalization of glycemic state, uneventful surgical excision of mass (measuring 36 mm × 26 mm × 21 mm) through a lateral orbitotomy approach was done [Fig. 2a, 2b] and the specimen was subjected to histological examination.

**Figure 3:**
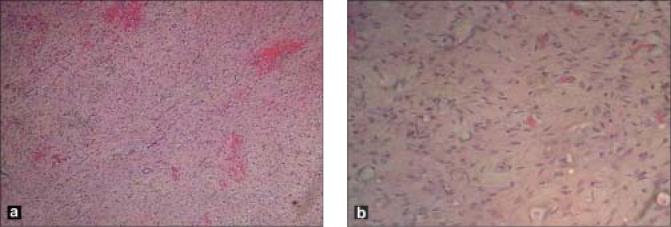
(a) Lower magnification (×10) shows spindle-shaped cells with uniform nuclei (b) higher magnification (×40) shows spindle-shaped cells with uniform nuclei containing finely dispersed chromatin. Dense collagen bundles are woven between the cells (H and E stain)

Hematoxylin and Eosin stain of multiple sections showed spindle cells arranged in hypercellular and hypocellular pattern with rich vascularity. Large numbers of capillaries containing erythrocytes were also noted in the periphery [Fig. 3a and 3b]. There was no evidence of mitotic figures or necrosis. With trichome staining, the tumor exhibited predominantly collagen production. Immunohistochemistry reactivity to CD34 confirmed the diagnosis of an orbital SFT. Subsequently, there was improvement in visual acuity from 20/120 to 20/20 in the right eye and normalization of IOP (14 mm Hg). No recurrence was noted over the two years of follow-up.

## Discussion

SFT are rare spindle-cell neoplasms normally found associated with serosal surfaces, especially the pleurae. Only recently, they have been recognized in extra-serosal sites such as lungs, liver, paranasal sinuses, salivary glands, adrenals, dural sheath and the orbit.[1,2] Diagnosis of SFT can only be performed by histological examination, as clinical signs and radiological features are not conclusive.[3]

The classic histopathological features of SFT such as thick bands of collagen, alternating hypercellular and hypocellular areas, and a hemangiopericytoma-like pattern of vascularity[4] are consistent with our findings.

Often from microscopic features alone it may be difficult to differentiate SFT from other spindle-shaped cell tumors of the orbit such as fibrous histocytoma, hemangiopericytoma, meningioma and Schwannoma. The reason behind the low incidence of orbital SFT is probably because it histologically mimics other spindle-cell tumors and hence was first reported only in 1994.[1]

The key to differentiating SFT is its immunohistochemical reactivity. SFT shows strong and diffuse positivity with vimentin, BCL2 and CD 34+.[5,6] CD34 is an antigen expressed on the surface of the endothelium and hematopoietic progenitor cells. CD34 immunoreactivity was consistently found in SFT and this marker facilitates histopathological differentiation of this lesion from other recognized spindle cell tumors of the orbit. SFT have demonstrated strong CD34+ reactivity in 79-100% of cases. In our case, immunohistochemical positivity to CD 34+ confirmed the diagnosis of SFT.

Though the association of orbital SFT with pregnancy is a rare association, the focal expression of progesterone receptors, in the tumor cells may be related to pregnancy.[7] Recently, it has been described that steroid hormone receptors, progesterone receptors in particular, are expressed by extra-pleural SFT. In addition, progesterone may participate as growth factor in many CD34(+) neoplasms, which expresses low levels of the hormone receptors.[8]

In our case, the rapid growth of the tumor during pregnancy adds another dimension to the behavior of the tumor and is probably due to the presence of hormone receptors in orbital SFT. Similar progression of cavernous hemangioma of the orbit in pregnancy might be misleading in the clinical diagnosis of this entity.
